# Molecular Characterization and Expression of the Ecdysone Receptor and Ultraspiracle Genes in the Wheat Blossom Midge, *Sitodiplosis mosellana*

**DOI:** 10.3390/insects16050537

**Published:** 2025-05-19

**Authors:** Qitong Huang, Linqing Meng, Yuhan Liu, Keyan Zhu-Salzman, Weining Cheng

**Affiliations:** 1 Shandong Institute of Sericulture, Jiaodong Innovation Center, Shandong Academy of Agricultural Sciences, Yantai 264002, China; 13760637249hqt@nwafu.edu.cn; 2 Key Laboratory of Plant Protection Resources and Pest Management of Ministry of Education, College of Plant Protection, Northwest A&F University, Yangling 712100, China; menglinqin@nwafu.edu.cn (L.M.); liuyuhannancy@163.com (Y.L.); 3 Department of Entomology, Texas A&M University, College Station, TX 77843, USA

**Keywords:** diapause, 20-hydroxyecdysone, *EcR*, *USP*, *Sitodiplosis mosellana*

## Abstract

20-hydroxyecdysone (20E) is essential for insect development and diapause. Ecdysone receptor (EcR) and ultraspiracle (USP) proteins are crucial regulators of 20E signaling. To explore their potential roles in the development of *Sitodiplosis mosellana*, a major wheat pest that undergoes obligatory diapause as a larva, one *SmEcR* and two *SmUSPs* (*SmUSP-A* and *SmUSP-B*) from this species were isolated and characterized. The deduced SmEcR and SmUSP-A/B proteins contained a conserved DNA-binding domain with two zinc finger motifs that bind to specific DNA sequences. Expression of *SmEcR* and the *SmUSPs* was developmentally controlled, as was 20E induction. Their transcription levels increased as the larvae entered pre-diapause, followed by downregulation during diapause and upregulation during the shift to post-diapause quiescence, which is highly consistent with ecdysteroid titers in this species. Topical application of 20E to diapausing larvae also elicited a dose-dependent expression of the three genes. Expression of *SmEcR* and *SmUSPs* decreased markedly during the pre-pupal stage and was higher in adult females compared to males. These findings suggested that 20E-induced expression of *SmEcR* and *SmUSPs* has key roles in diapause initiation and maintenance, post-diapause quiescence, and adult reproduction, while the larval–pupal transformation may be associated with a decrease in their expression levels.

## Simple Summary

20-hydroxyecdysone (20E) has a major role in the regulation of development and diapause in insects. Ecdysone receptor (EcR) and ultraspiracle (USP) proteins are primary regulators involved in 20E signaling. However, the functions of these genes during diapause are poorly understood. This study aimed to characterize the genes encoding EcR and USP (*SmEcR*, *SmUSP-A*, and *SmUSP-B*) in *Sitodiplosis mosellana*, a major wheat pest that undergoes obligatory diapause during the mature third larval instar, and analyze their expression dynamics across various developmental stages, as well as their responsiveness to 20E treatment. The results showed that the expression of *SmEcR*, *SmUSP-A*, and *SmSP-B* was not only developmentally controlled but also responsive to 20E. The transcription levels of the genes increased as the larvae entered pre-diapause, followed by downregulation during diapause and upregulation during transition to post-diapause quiescence, with higher expression observed in adult females compared to males. These results suggest that 20E-responsive *SmEcR* and *SmUSP* genes mediate critical functions during diapause initiation, maintenance, and post-diapause quiescence, and also contribute to adult reproduction, while the larval–pupal transition may be associated with a downregulation of their expression.

## 1. Introduction

Insects are the most successful animal group in terms of species number. Although multiple factors contribute to this profusion, one of the most important is the characteristic insect developmental plan of metamorphosis and diapause. Diapause is a genetically determined state of developmental arrest, which permits insects to survive in adverse environmental conditions and also to synchronize their life cycle with seasons favorable for development and reproduction [1,2]. Due to the central role of diapause in life-cycle synchronization and adaptation to environmental extremes, an in-depth understanding of the molecular mechanism underlying the process is essential for the development of effective pest management strategies [3].

Steroid hormone 20-hydroxyecdysone (20E) is known to modulate many important aspects of insect life, including not only molting, metamorphosis, and reproduction [4,5,6], but also diapause. It has been demonstrated that in some insects undergoing larval diapause, a drop in or low level of 20E titer is associated with the initiation and maintenance of diapause, while a rise in 20E or its exogenous application can terminate diapause, such as the bamboo borer *Omphisa fuscidentalis* [7,8], the corn earworm *Helicoverpa zea* [9], the peach fruit moth *Carposina sasakii* [10], and the cabbage white butterfly *Pieris rapae* [11]. The effects of 20E are mediated through two nuclear receptors: the ecdysone receptor (EcR) and its heterodimeric partner ultraspiracle (USP), a homolog of the vertebrate retinoid X receptor (RXR) [12,13,14]. The EcR/USP heterodimer binds to ecdysone response elements (EcREs) within the promoter regions of 20E-responsive genes, triggering transcriptional cascades that drive developmental transitions [15,16,17].

The genes encoding EcR and USP have been identified and characterized in a variety of insect species. Three EcR isoforms, namely, EcRA, EcRB1, and EcRB2, which have different N-terminal regions but the same DNA- and hormone-binding domains, were identified in *Drosophila melanogaster* [18], while one or two isoforms of EcR have been observed in the majority of other investigated insects, including *Bombyx mori*, *Diaphorina citri*, *Leptinotarsa decemlineata*, and *Monochamus alternates* [19,20,21,22,23]. In contrast to EcR, only one USP form has been found in *Drosophila*, while two isoforms with unique N-termini are present in many other species [24,25,26]. Recent studies have investigated the functions of EcR and the USP isoforms in the control of insect metamorphosis and molting [27,28,29]. However, to date, little information is available about their relationship with larval diapause [30,31].

The wheat blossom midge *Sitodiplosis mosellana* Géhin (Diptera: Cecidomyiidae), a major wheat pest, is widely distributed in the main wheat-producing regions of the northern hemisphere [32,33,34]. This species is univoltine with an obligatory larval diapause. In most areas of northern China, adult emergence and subsequent oviposition on wheat heads typically occur from mid- to late April [35]. The hatched larvae feed on the developing kernels until the larvae mature to the third instar. Matured larvae fall to the ground in mid-to-late May, spinning cocoons in the soil to initiate diapause. While the larvae remain in the cocoons until mid-March of the following year, diapause is concluded at the onset of cold weather in early December, after which the larvae enter post-diapause quiescence [36], which is morphologically identical to diapause. Post-diapause development is initiated by increased ambient temperature in spring, with the larvae leaving their cocoons and pupating on the soil surface. Therefore, the prolonged diapause and quiescence enable this species to combat harsh environmental conditions during summer and winter, and also synchronize its developmental cycle with that of its wheat host.

Previous studies have suggested the involvement of 20E in regulating the *S. mosellana* diapause [37], although the underlying molecular mechanism remains unclear. To explore the molecular basis of 20E effects on *S. mosellana*, the present study cloned and characterized cDNAs encoding EcR and USP from this species, namely, *SmEcR*, *SmUSP-A*, and *SmUSP-B*. The dynamic expression of these genes at different stages of diapause and direct development was also investigated, and the effects of topical application of 20E on expression were examined. The results of this study provide a foundation for further investigation into the functions of these genes in regulating the development of *S. mosellana*, assisting in the development of novel control strategies for this major pest.

## 2. Materials and Methods

### 2.1. Experimental Insects

Samples of *S. mosellana* at all developmental stages except the egg were collected from a natural setting using our previously developed method [38]. Briefly, the first two instars and pre-diapause third instar larvae, which were differentiated by body size, coloration, and the sternal Y-shaped structure [39], were obtained during May 2022 by dissecting wheat ears at the grain filling stage that were infested with *S. mosellana*. Concurrently, wheat ears containing 3rd instar larvae were collected in bulk and placed on soil in a field insectary established in Yangling, China (34°16′ N, 108°4′ E). The ears were sprayed with water to induce larval egress from the glumes and their subsequent migration into the soil. Successful initiation of diapause was confirmed by the presence of larval cocoons. Cocooned larvae, including those in the diapause (June–November) and post-diapause quiescent stages (December–February) [36] were harvested by sieving the soil in the insectary monthly, from 25 June 2022 to 25 February 2023. Post-diapause larvae (i.e., larvae exiting cocoons), pre-pupae, early-to-late-stage pupae, and adults were subsequently collected from mid-March. Groups of 50 1st and 2nd instar larvae or 20 individuals from each developmental stage were placed in 1.5 mL tubes, with at least three replicates from each sample. The samples were snap-frozen in liquid nitrogen and subsequently maintained at −80 °C for later use.

### 2.2. Cloning of S. mosellana EcR and USP Genes

Total RNA was extracted from 20 homogenized 3rd instar larvae using RNAiso Plus reagent (TaKaRa, Dalian, China) as per the manufacturer’s instructions. The concentration and purity of the RNA were assessed spectrophotometrically by the absorbance ratio at 260/280 nm (i.e., OD_260_/OD_280_), and the integrity was confirmed by 1% agarose gel electrophoresis. First-strand complementary DNA (cDNA) was synthesized from 1 μg of purified RNA using the PrimeScript^TM^ RT reagent Kit with gDNA Eraser (TaKaRa, Dalian, China), following the provided instructions, and preserved at −20 °C until use.

An EcR sequence and two USP isoforms (i.e., USP-A and USP-B) were identified in an *S. mosellana* transcriptomic dataset established in our laboratory and a genomic database [40] using similar gene sequences from the copepod *Tigriopus japonicus* [41]. Specific primers (Table 1) for amplification of the open reading frames (ORFs) were designed and synthesized. The PCR reactions (in 20 μL) consisted of 10 μL of 2×Taq MasterMix (CWbiotech, Beijing, China), 1 μL of cDNA, 0.5 μL of each primer (10μM), and 8 μL of ddH_2_O. The amplification conditions were an initial denaturation at 94 °C for 3 min, followed by 30 cycles (95 °C, 30 s), annealing (55 °C, 30 s), and extension (72 °C, 60 s for USPs or 90 s for EcR), concluding with a 10 min final extension at 72 °C. The PCR amplicons were purified from gels and then ligated into the pMD19-T vector. Recombined plasmids were transformed into DH5α-competent *E. coli* cells and sequenced.

### 2.3. Sequencing and Phylogenetic Analysis

The molecular weights and theoretical isoelectric points of EcR, USP-A, and USP-B proteins from *S. mosellana* were determined using the Compute pI/Mw tool (http://web.expasy.org/protparam, accessed on 15 May 2023). Functional domains and motifs were identified using a conserved domain search tool (http://www.ncbi.nlm.nih.gov/Structure/cdd/wrpsb.cgi, accessed on 15 May 2023) and the SMART tool (http://smart.embl-heidelberg.de/, accessed on 15 May 2023). Multiple sequence alignments of the SmEcR and SmUSP proteins with those from other insect species were performed using DNAMAN 6.0 software (Lynnon Corporation, Pointe-Claire, QC, Canada). A neighbor-joining phylogenetic tree was constructed [42] using MEGA7 with 1000 bootstrap repeats.

### 2.4. 20E Treatment

To examine the effects of 20-hydroxyecdysone (20E) on the expression of *S. mosellana EcR* and *USP* genes in diapause larvae, 20E was diluted in 50% ethanol to concentrations of 0.1–0.6 pg/nL. These solutions (23 nL) were injected using a Nanoject Ⅱ Auto-Nanoliter Injector (Drummond Scientific Company, Broomall, PA, USA) into the abdomens of cocooned larvae collected in October. The doses of 20E applied were based on the highest reported levels of endogenous 20E in the larvae of this insect [37]. The treated larvae were placed in petri dishes with moist filter paper and incubated for 1, 3, or 6 h at 25 °C in environmental incubators with 70% relative humidity before being frozen in liquid nitrogen and stored at −80 °C for subsequent RNA isolation. Insects treated with the same volume of 50% ethanol acted as the controls. Each treatment was performed using at least three replicates with 20 individuals per replicate.

### 2.5. Expression Analysis Using Reverse-Transcription Quantitative PCR (RT-qPCR)

To investigate the expression patterns of the *S. mosellana EcR* and *USP* genes during diapause and metamorphosis, approximately 50 1st instar larvae, 50 2nd instar larvae, and 20 3rd instar larvae at the different developmental stages related to diapause, as well as 20 pupae at different developmental stages, and adults, were used for RNA extraction. To examine the transcript levels in 20E-treated larvae, 20 individuals from each treatment group were used for RNA extraction. RNA was extracted using the RNAiso Plus reagent (TaKaRa, Dalian, China). The template cDNA was prepared from 1 μg of RNA with the PrimeScript^TM^ RT reagent Kit with gDNA Eraser (TaKaRa, Dalian, China).

RT-qPCR was conducted on an ABI 7500 real-time PCR system (Applied Biosystems, Foster City, CA, USA) (Thermofisher, Waltham, MA, USA) in a 20 μL reaction volume containing 2 μL of template cDNA, 10 μL of 2×SYBR Green Premix EX TaqTM II (TliRNaseH Plus) (TIANGEN, Beijing, China), 0.8 μL each of forward and reverse 10 μM primers (Table 1), 0.4 μL of 50×ROX Reference Dye, and 6.0 μL of ddH_2_O. *GAPDH* (GenBank accession number: KR733066) was used as an endogenous reference. The amplification procedure was performed using the two-step method, with an initial pre-denaturation at 95 °C for 30 s, followed by 40 cycles of denaturation (95 °C, 10 s), and annealing (60 °C, 30 s). The amplification specificity was verified through melting curve analysis with temperature gradients of 95 °C for 5 s, 60 °C for 1 min, and 95 °C for 15 s. The negative controls used RNase-free water instead of the cDNA template. Standard curves were generated using serially diluted pooled cDNA samples for target genes and *GAPDH*. The amplification efficiencies for primers of all genes were similar and close to 100%. Each treatment was performed in triplicate, with technical triplicates per sample. The cycle threshold value (Ct) of each target gene was normalized to that of *GAPDH* in the different samples, and relative expression was calculated using the 2^−∆∆CT^ method [43].

### 2.6. Statistical Analysis

Data are reported as means and standard error (SE) from three independent replicates per sample. Duncan’s multiple range test was used with analysis of variance (ANOVA) to compare differences between samples and treatments, with *p* < 0.05 considered statistically significant. All statistical computations were conducted in SPSS 22.0 software (SPSS Inc., Chicago, IL, USA).

## 3. Results

### 3.1. Characterization of SmEcR and SmUSP cDNAs

The complete open reading frame (ORF) of *S. mosellana EcR* (*SmEcR*) cloned by PCR was found to be 1563 bp in length (Figure S1, accession No. MN853890), encoding a protein with 520 amino acids, a molecular weight (MW) of 59.6 kDa, and an isoelectric point (pI) of 5.51. The ORFs of the *S. mosellana* USP isoforms, *SmUSP-A* (Figure S2, accession No. MN853891) and *SmUSP-B* (Figure S2, accession No. MN853892), were 1377 and 1314 bp, respectively. These encoded proteins of 458 and 437 amino acids have estimated MWs of 51.6 and 49.2 kDa, and pI values of 9.00 and 8.78, respectively.

**Figure 1:**
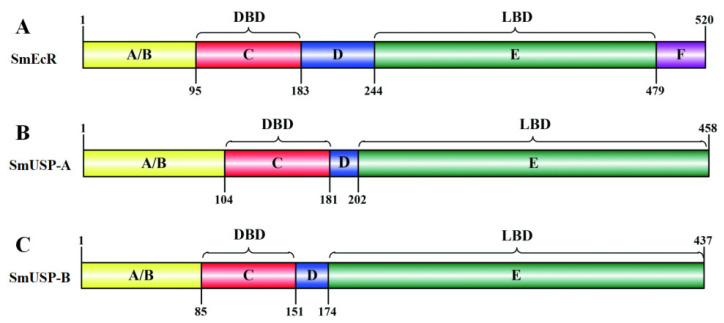
Schematic representation of SmEcR (**A**), SmUSP-A (**B**), and SmUSP-B (**C**). Functional domains of the coding region are visually depicted by distinct color-coded boxes: A/B domain (transactivation domain, yellow), C domain (DNA binding domain, DBD, red), D domain (hinge domain, blue), E domain (ligand binding domain, LBD, green), and F domain (purple).

**Figure 2:**
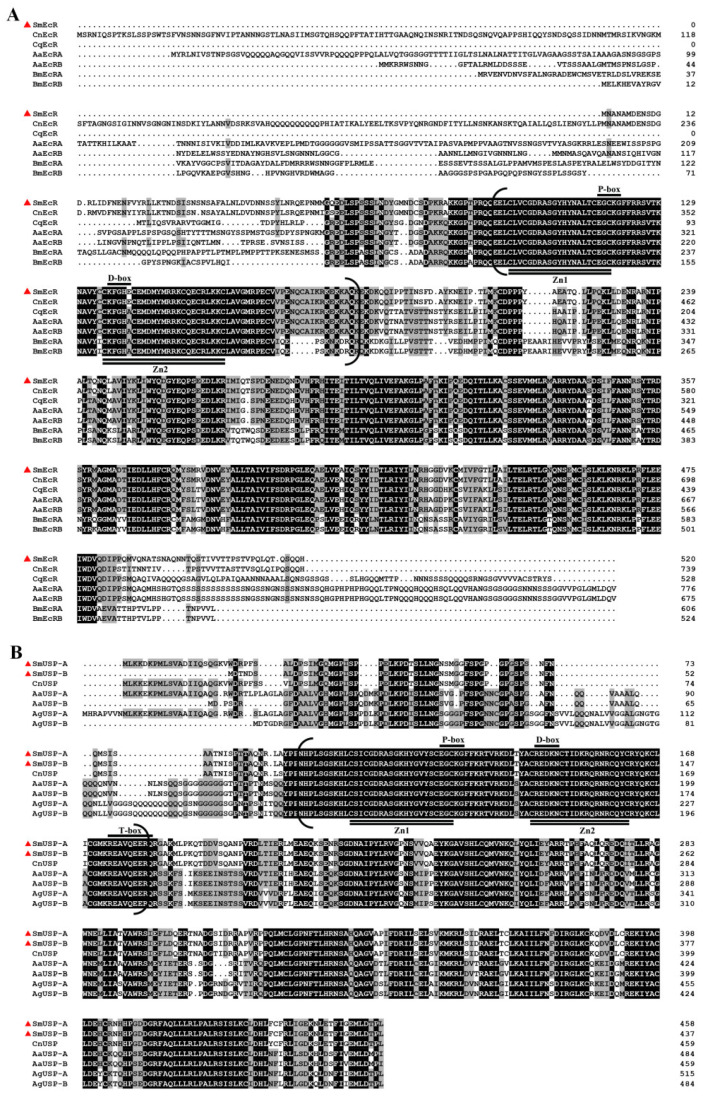
Sequence alignments of *Sitodiplosis mosellana* EcR (SmEcR) (**A**) and USP (SmUSP-A and SmUSP-B) (**B**) proteins in relation to their homologs from other insect species. SmEcR and SmUSPs are indicated with red triangles. Identical and similar amino acids were emphasized through black and grey shading, respectively. DNA-binding domain is denoted by brackets. Two zinc-finger motifs are emphasized with double underlines. The P-box (E117-G121 for SmEcR, E125-G129 for USP-A, and E104-G108 for USP-B), D-box (K136-G140 for SmEcR, R144-N148 for USP-A, and R123-N127 for USP-B), and T-box (R174-Q182 for USP-A and R153-Q161 for USP-B) sequences were underscored with underlines. Species abbreviations: Sm, *Sitodiplosis mosellana*; Cn, *Contarinia nasturtii*; Aa, *Aedes aegypti*; Bm, *Bombyx mori*; Ag, *Anopheles gambiae*.

Domain searching showed that SmEcR and two SmUSPs shared several typical structural hallmarks of the nuclear hormone receptor superfamily. These were N-terminal A/B (transactivation domain), C (DNA binding domain, DBD), D (hinge domain), and E (ligand binding domain, LBD) domains; however, SmEcR contained an additional F domain (Figure 1A,C) [44]. Further, two highly conserved C_2_C_2_ Zn-finger motifs, a P-box (EGCKG for SmEcR and SmUSPs) and a D-box (KFGHE for SmEcR and REDKN for SmUSPs), which are responsible for the recognition and binding to the hormone response elements (HREs) in target genes [24,45], were identified in the DBD of three putative proteins; while a T-box sequence (REAVQEERQ), also a DNA-recognition motif, was discovered in the D domain of two SmUSPs (Figure 2A,B).

### 3.2. Sequence Comparison and Phylogenetic Analyses

A comparison of the deduced amino acid sequences showed that SmEcR had the highest sequence identity (92%) with EcR from *Contarinia nasturtii* (XP_031622281.1), and 86% identity to EcRs from *Culex quinquefasciatus* (EDS37702.1) and *Aedes aegypti* (XP_021710216.1, XP_021710217.1). In contrast, SmEcR showed less than 67% identity with *Bombyx mori* EcR (XP_021206378.1, NP_001166846.1) (Figure 2A). Similarly, SmUSP-A (QOE77677.1) and SmUSP-B (QOE77678.1) exhibited the highest identity with *C. nasturtii* USP (XP_031630693.1), achieving 98% and 92% identity, respectively, and 62–67% identity with USPs from *Aedes aegypti* (AAEL000395-PA, AAEL000395-PB) and *Anopheles gambiae* (AGAP002095-PD, AGAP002095-PC). The two SmUSPs were 94% identical (Figure 2B).

**Figure 3:**
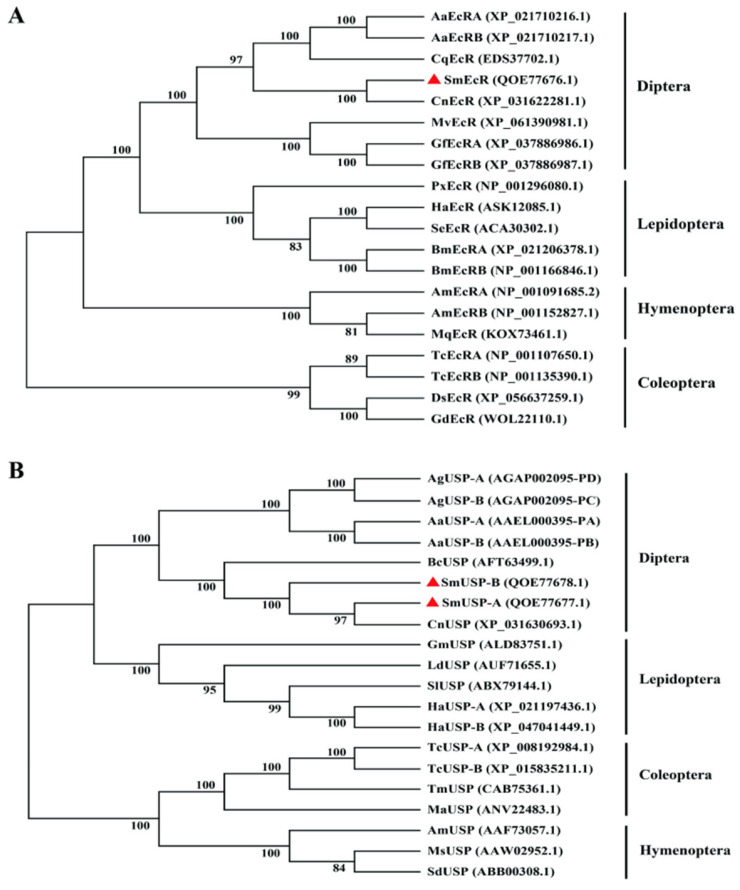
Phylogenetic neighbor-joining tree of *Sitodiplosis mosellana* EcR (SmEcR) (**A**) and USP (SmUSP-A and SmUSP-B) (**B**) proteins in relation to their homologs from other insect species. SmEcR and SmUSPs are indicated with red triangles. Species abbreviations: Sm, *Sitodiplosis mosellana*; Cn, *Contarinia nasturtii*; Aa, *Aedes aegypti*; Bm, *Bombyx mori*; Cq, *Culex quinquefasciatus*; Mv, *Musca vetustissima*; Gf, *Glossina fuscipes*; Px, *Plutella xylostella*; Ha, *Helicoverpa armigera*; Se, *Spodoptera exigua*; Am, *Apis mellifera*; Mq, *Melipona quadrifasciata*; Tc, *Tribolium castaneum*; Ds, *Diorhabda sublineata*; Gd, *Galeruca daurica*; Ag, *Anopheles gambiae*; Bc, *Bradysia coprophila*; Gm, *Grapholita molesta*; Ld, *Lymantria dispar*; Sl, *Spodoptera litura*; Tm, *Tenebrio molitor*; Ma, *Monochamus alternatus*; Ms, *Melipona scutellaris*; and Sd, *Scaptotrigona depilis*.

As anticipated, phylogenetic analysis revealed distinct clustering of EcRs and USPs derived from Dipteran, Lepidopteran, Coleopteran, and Hymenopteran insects, corresponding to their established taxonomic divergence. Notably, SmEcR and SmUSP-A/B were more closely related to their counterparts in the dipteran Cecidomyiidae family (*C. nasturtii*), emphasizing their close phylogenetic ties (Figure 3A,B).

### 3.3. Expression of SmEcR and SmUSPs During Diapause

**Figure 4:**
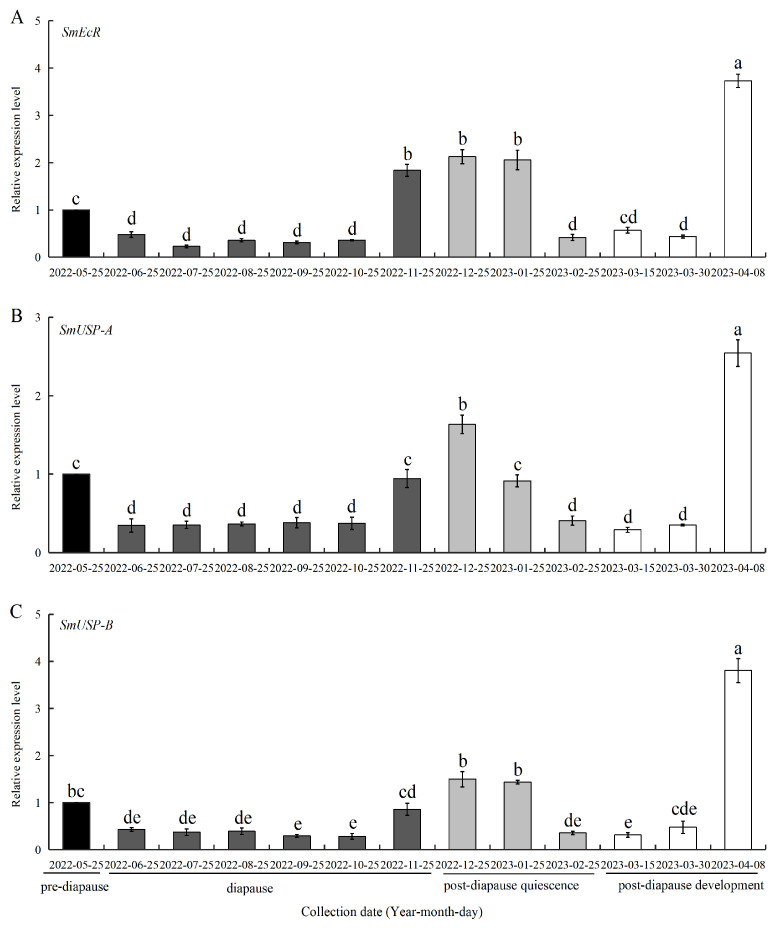
Relative mRNA levels of *Sitodiplosis mosellana EcR* (**A**), *USP-A* (**B**), and *USP-B* (**C**) in pre-diapause, diapause, and post-diapause larvae. The relative transcript abundance (means ± SE) of all tested stages was normalized against the pre-diapause larvae, which served as a calibrator with an assigned value of 1. Different letters above the bars indicated significant differences using Duncan’s multiple range test after ANOVA (*p* < 0.05).

The expression profiles of *SmEcR* and the *SmUSPs* were determined in four successive diapause processes of *S. mosellana*; specifically, 3rd instar larvae, including the pre-diapause, diapause, post-diapause quiescence, and post-diapause developmental stages, as shown in Figure 4. *SmEcR* expression was downregulated upon entry into diapause (June), with low expression throughout diapause (June-October), but was significantly upregulated from late November, when most *S. mosellana* end diapause and enter post-diapause quiescence [36], after which expression remained constant until the mid-quiescent period (January). Expression then dropped sharply, falling back to the level at the early-to-mid period of post-diapause development (mid-to-late March). Thus, the highest expression was observed during the late developmental stage (early April) (Figure 4A).

Similarly, the expression levels of *SmUSP-A* and *SmUSP-B* were low during diapause and the early-to-mid stage of post-diapause development, peaking at the late stage of post-diapause development, which was then the quiescent period in December. In contrast to *SmEcR*, whose expression during early-to-mid quiescence (November–January) was stable and significantly higher than that at pre-diapause (May), the levels of *SmUSP-A* and *SmUSP-B* expression were both higher in December than those in November and January, with the November and January levels similar to those observed again during the mid-to-late period of post-diapause development (Figure 4B,C).

### 3.4. 20E Regulation of SmEcR and SmUSP Genes During Diapause

**Figure 5:**
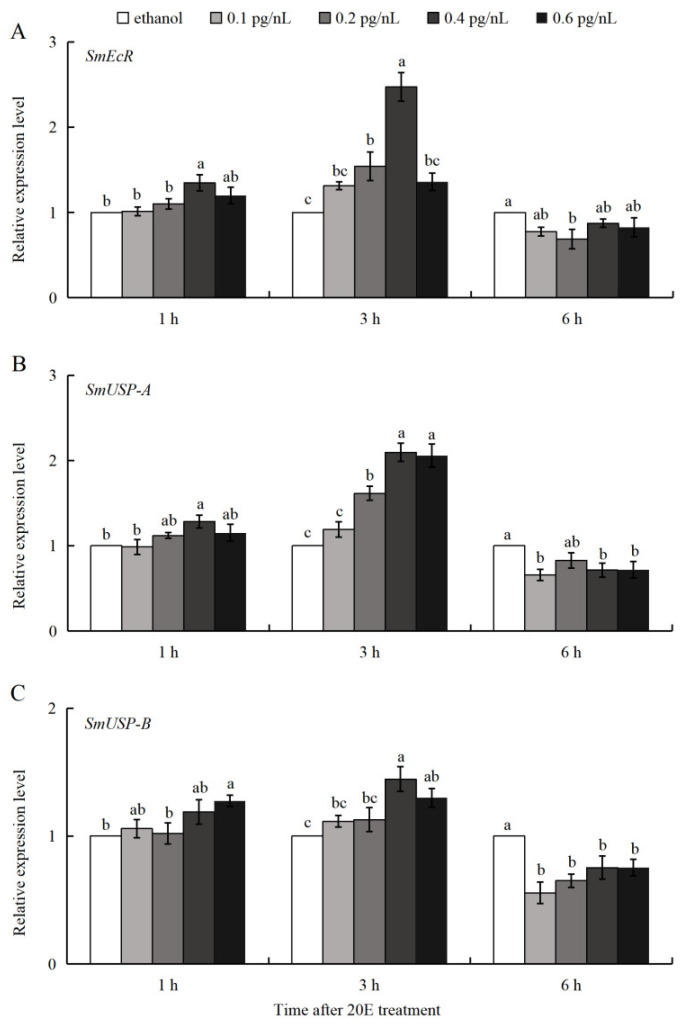
Relative mRNA levels of *Sitodiplosis mosellana EcR* (**A**), *USP-A* (**B**), and *USP-B* (**C**) at 1, 3, and 6 h time points after 20E injections at concentrations spanning 0.1 to 0.6 pg/nL into diapausing larvae. The relative transcript abundance (means ± SE) of each treatment was normalized against the ethanol control, which served as a calibrator with an assigned value of 1. Different letters above the bars within each time point indicated significant differences using Duncan’s multiple range test after ANOVA (*p* < 0.05).

Treatment of larvae in diapause with 20E (0.1–0.6 pg/nL) resulted in significant upregulation of *SmEcR* and *SmUSP-A* 1 h after treatment with 0.4 pg/nL 20E and 3 h after treatment at concentrations of 0.2–0.6 pg/nL for *SmEcR* and 0.2–0.4 pg/nL for *SmUSP-A*. The maximum expression values were observed at 3 h after treatment with 0.4 pg/nL 20E for *SmEcR* and *SmUSP-A*, with 2.5-fold and 2.1-fold increases relative to the control, respectively (Figure 5A,B). The expression of *SmUSP-B* only increased significantly at 3 h after treatment with 0.4 pg/nL (1.5-fold) and 0.6 pg/nL (1.3-fold) (Figure 5C). Notably, the expression of all three genes was downregulated for all concentrations at the 6 h time point after treatment.

### 3.5. Expression of SmEcR and SmUSP Genes at Different Developmental Stages

**Figure 6:**
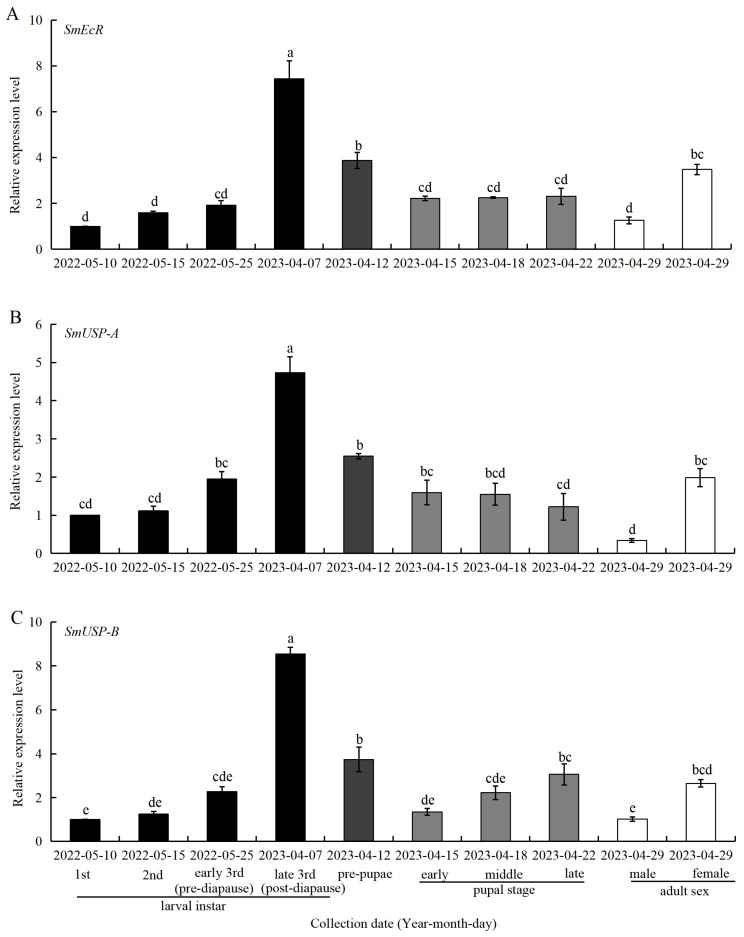
Relative expression levels of *Sitodiplosis mosellana EcR* (**A**), *USP-A* (**B**), and *USP-B* (**C**) in different developmental stages. The relative transcript abundance (means ± SE) of each stage was normalized against 1st instar larvae, which served as a calibrator with an assigned value of 1. Different letters above the bars indicated significant differences using Duncan’s multiple range test after ANOVA (*p* < 0.05).

The expression profiles of *SmEcR*, *SmUSP-A*, and *SmUSP-B* were also determined across all developmental stages of *S. mosellana*, including larval instars (1st, 2nd, early-3rd, and late-3rd instar larvae), pre-pupae, and pupae (early, middle, and late-stage pupae), as well as adults (males and females) (Figure 6).

The expression profiles of the three genes varied significantly among the different developmental stages (*p* < 0.05), with the highest expression levels seen in the late-3rd instar larvae. Marked reductions in expression were observed in the transition to the pre-pupal stage, with further declines on development into pupae. The expression of *SmEcR* remained unchanged during the pupal stage, while that of *SmUSP-A* decreased gradually and that of *SmUSP-B* increased gradually. Notably, the expression of all three genes was significantly higher in females than in male adults.

## 4. Discussion

As functional ecdysone receptors, EcR and USP are considered pivotal in 20E-mediated larval diapause. However, the functions of these proteins during larval diapause are less well understood. In this study, we identified and characterized *EcR* and *USP* genes (*SmEcR*, *SmUSP-A*, and *SmUSP-B*) from *S. mosellana*, a significant wheat pest that undergoes typical obligatory larval diapause, and explored their expression profiles throughout the insect life cycle, as well as their responsiveness to 20E treatment. In contrast to many molecular investigations of insect development, all insect samples utilized in this study were obtained directly from field collections, potentially mirroring natural conditions more accurately.

Similar to the findings of previous studies, the identified SmEcR and SmUSPs contained the canonical structural domains of the nuclear receptor superfamily (Figure 1) [46,47]. In particular, the DNA-binding domains (DBDs), characterized by two zinc-finger motifs containing a proximal P-box and D-box sequences, were found to be highly conserved in the three putative proteins (Figure 2A,B). The P-box mediates base-specific recognition of hormone response element half-sites [48], whereas the D-box stabilizes homo-/heterodimer formation via hydrophobic packing [49], a mechanism co-opted in *Drosophila* to mediate EcR/USP transcriptional activity during metamorphosis [50], suggesting a conserved role for SmEcR/SmUSP in stage-specific gene regulation. Notably, the two SmUSP proteins also contained a T-box within the DBD domain (Figure 2B), which is crucial in mediating response element recognition via monomeric interactions with cognate sequences [44,51].

Deficiencies in 20E are documented to attenuate ecdysis-triggering hormone signaling and suppress the synthesis of juvenile hormone (JH), ultimately triggering diapause in *Colaphellus bowringi* [6]. Conversely, elevated concentrations of 20E promote diapause termination in *Bactrocera minax* and *Helicoverpa zea* by inducing the expression of *EcR* [9,52]. The potential roles of *SmEcR* and *SmUSPs* in the sensing and relaying of 20E signals during larval diapause were supported by the observed correspondence between gene expression patterns (Figure 4) and ecdysteroid titers [37], as well as the responsiveness of these genes to 20E induction (Figure 5). Notably, elevated expression of *SmEcR* and the *SmUSP* genes was observed in the early period of post-diapause quiescence (December), characterized by identical morphology to diapause [36]. At this stage, *S. mosellana* shows increased biosynthesis of both JH and ecdysteroid [37,53]. Presumably, the stage of larval quiescence state arises from the synergistic actions of ecdysteroid and JH, wherein ecdysteroid triggers diapause termination and growth by regulating the expression of *SmEcR* and the *SmUSPs*, counteracted by the antagonistic effect of JH mediated via *SmMet* and *SmKr-h1* [54]. These findings suggest that the diapause of *Sitodiplosis mosellana* is related to the expression of *EcR* or *USPs*. Therefore, future pest management strategies that focus on regulating diapause by interfering with the expression of these genes may provide a means to break their diapause characteristics and reduce the accumulation of pest sources, thereby promoting effective pest control of this major pest.

It is generally believed that a conspicuous drop in JH titer coupled with a sharp increase in ecdysteroid levels during the last instar larval stage of holometabolous insects heralds the imminent transformation from larva to pupa [55,56,57]. During this period, 20E actions are mediated by the EcR and USP heterodimer [14]. In the melon fly, *Zeugodacus cucurbitae*, *EcR* expression in the penultimate (second) and final (third) instar larvae was found to be substantially higher than that in the pupae, and its suppression halted the larval–pupal transition [58]. Similarly, *Grapholita molesta* exhibited heightened expression of *USP* prior to the final larval molt and during the nascent pupal stage [59]. In the current study, the expression of *S. mosellana EcR* and *USPs* in the late last instar larval stage was significantly higher than that in the pre-pupae and pupae (Figure 6A,C), echoing the significant elevations in ecdysteroid titers seen before pupation and the concomitant reduction in the expression levels of the JH receptor *SmMet* and its downstream transcription factor *SmKr-h1* [37,54]. The temporal expression patterns of *SmEcR* and *SmUSPs* are indicative of their pivotal role in the progression of larval and pupal development. Interestingly, the expression of *SmUSP-A* gradually decreased from the primary pupa stage to the postpupal stage, but *SmUSP-B* gradually increased (Figure 6B,C). Previous studies have shown that reduced *USP* expression in *Apis mellifera* may trigger the developmental pathways that stabilize cuticle formation by inhibiting the formation of the ecdysone receptor complex [60], whereas *USP* expression in *Drosophila melanogaster* was associated with stage-specific transcriptional regulation and its synergistic interaction with EcR [61]. Presumably, the opposite expression patterns of *SmUSP-A* and *SmUSP-B* in the pupal stage suggest potential antagonistic or complementary roles in regulating pupal development, though their functional divergence remains unresolved.

The results of recent studies have also implicated EcR and USP in the regulation of insect reproduction. For instance, inhibiting the expression of *EcR* and *USP* in female adults of the melon aphid, *Aphis gossypii*, and the desert locust, *Schistocerca gregaria*, reduces both insect chorion formation and oviposition, thereby markedly decreasing their reproductive capacity [46,62]. Suppression of *EcR* and *USP* expression in the adult female citrus psyllids, *Diaphorina citri*, was found to significantly impede ovarian maturation and curtail egg output [23]. The observed higher expression of *EcR* and the *USPs* in female *S. mosellana* adults compared to their male counterparts (Figure 6) suggests the potential involvement of these genes in the reproductive process.

## 5. Conclusions

In summary, the results of the present study shed light on the molecular characterization of *S. mosellana* EcR and USP proteins, ecdysone-responsive transcription factors involved in 20E-mediated larval diapause and adult reproduction. A deeper understanding of their regulatory mechanisms may offer novel strategies for the effective management of this insect pest.

## Figures and Tables

**Table 1:** Primer sequences used in this study.

Primer Name	Sequence (5′ to 3′)	Purpose
EcR sense	TCGTGGCTCCGATTTTAT	ORF cloning
EcR antisense	GCAACGAATCTTACGGAT	
USP-A sense	TGAACGAGCGACACTGAA	
USP-A antisense	CACCCCAATCACTGTCAA	
USP-B sense	CGAGCAGATAAACCTACG	
USP-B antisense	CTGGTGTGACTATCTGGC	
EcR sense	TGCGGCGTAAGTGTCAGGA	qPCR
EcR antisense	GCTTTCTTCTCTTTGCGTTTGA	
USP-A sense	AATGCTTTCCGTAGCCGATATA	
USP-A antisense	CTGGTTTGAGTTCAGGCGGT	
USP-B sense	ATGGATACAAATGATTCAGCTTTGG	
USP-B antisense	TGGTTGAAATTGCTGGGGC	
GAPDH sense	CCATCAAAGCAAGCAAGA	
GAPDH antisense	CAGCACGGAGCACAAGAC	

## Data Availability

The original contributions presented in this study are included in the article/Supplementary Materials. Further inquiries can be directed to the corresponding authors.

## Supplementary Materials

The following supporting information can be downloaded at https://www.mdpi.com/article/10.3390/insects16050537/s1. Figure S1. Nucleic acid and predicted amino acid sequences of *SmEcR* in *Sitodiplosis mosellana*. Start codon (ATG) and stop codon (TAG) were marked with an ellipse. A/B, C, D, E and F domains are indicated by shadows. Two zinc-finger motifs “CLVCGDRASGYHYNALTCEGC” and “CKFGHECEMDMYMRRKCQECRLKKC” are emphasized with underlines. The P-box (EGCKG) and D-box (KFGHE) are boxed; Figure S2. Nucleic acid and predicted amino acid sequences of *SmUSP-A* and *SmUSP-B* in *Sitodiplosis mosellana*. Start codon (ATG) and stop codon (TAA) were marked with an ellipse. A/B, C, D and E domains are indicated by shadows. Two zinc-finger motifs “CSICGDRASGKHYGVYSCEGC” and “CREDKNCTIDKRQRNRCQYC” are emphasized with underlines. The P-box (EGCKG), D-box (REDKN) and T-box (REAVQEERQ) are boxed.
