# Supplementary material for: Molecular Characterization and Expression of the Ecdysone Receptor and Ultraspiracle Genes in the Wheat Blossom Midge, Sitodiplosis mosellana
Source: Insects. 2025 May 19;16(5):537. doi: 10.3390/insects16050537 (PMC12111886; doi:10.3390/insects16050537)
Supplement: Supplementary file 1 [file insects-16-00537-s001.zip › insects-3631712-supplementary.pdf]

insects
MDPI

Supplementary Materials: The following are available online. Figures S1 and S2

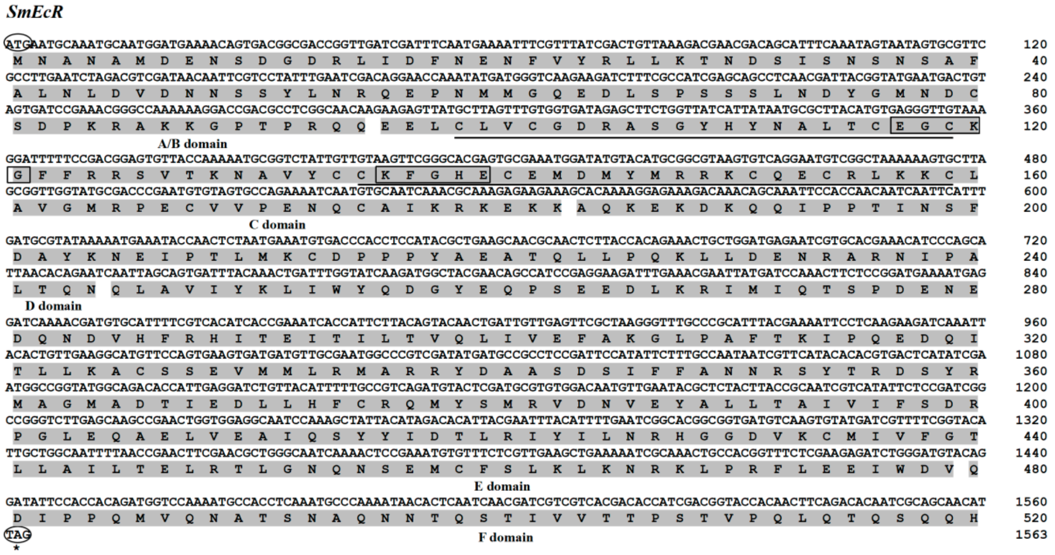

Figure S1. Nucleic acid and predicted amino acid sequences of *SmEcR* in *Sitodiplosis mosellana*. Start codon (ATG) and stop codon (TAG) were marked with an ellipse. A/B, C, D, E and F domains are indicated by shadows. Two zinc-finger motifs "CLVCGDRASGYHYNALTCEGC" and"CKFGHECEMDMYMRRKCQECRLKKC"are emphasized with underlines. The P-box (EGCKG) and D-box (KFGHE) are boxed.

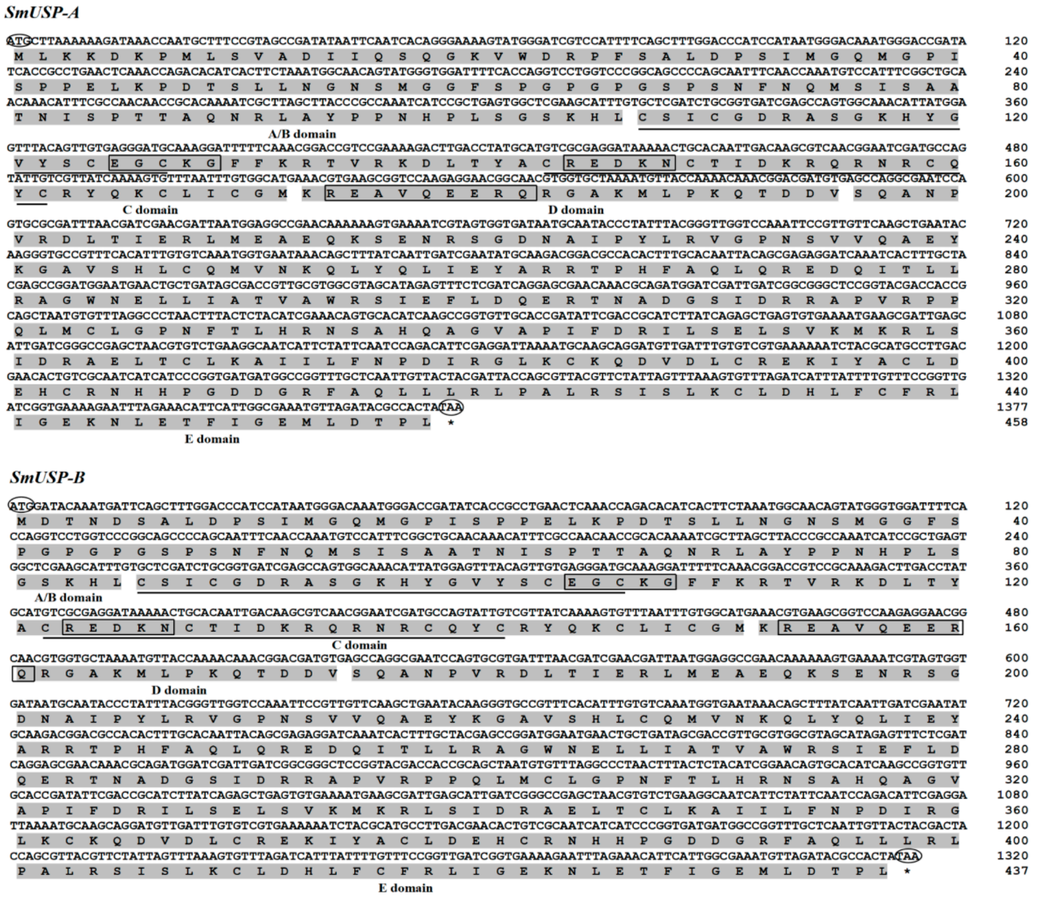

Figure S2. Nucleic acid and predicted amino acid sequences of *SmUSP-A* and *SmUSP-B* in *Sitodiplosis mosellana*. Start codon (ATG) and stop codon (TAA) were marked with an ellipse. A/B, C, D and E domains are indicated

by shadows. Two zinc-finger motifs“CSICGDRASGKHYGVYSCEGC”and“CREDKNCTIDKRQRNRCQYC”are emphasized with underlines. The P-box (EGCKG), D-box (REDKN) and T-box (REAVQEERQ) are boxed.
